# Engineering of a Novel Amphibian Skin Peptide Isolated from Agua Rica Leaf Frog (*Callimedusa ecuatoriana*) into Active Antimicrobial Agents

**DOI:** 10.3390/antibiotics14121186

**Published:** 2025-11-21

**Authors:** Stefanny Bonilla-Jiménez, Nina Espinosa de los Monteros-Silva, Giovanna Morán-Marcillo, Sebastián Bermúdez-Puga, Andrea Terán-Valdez, José R. Almeida, Carolina Proaño-Bolaños

**Affiliations:** 1 Biomolecules Discovery Group, Universidad Regional Amazónica Ikiam, km 7 ½ vía Muyuna, Tena 150150, Ecuador; stefanny.bonilla@epn.edu.ec (S.B.-J.); sebastianbermudez@usp.br (S.B.-P.); rafael.dealmeida@ikiam.edu.ec (J.R.A.); 2 Laboratory of Molecular Biology and Biochemistry, Universidad Regional Amazónica Ikiam, km 7 ½ vía Muyuna, Tena 150150, Ecuador; nina.espinosadelosmonteros@ikiam.edu.ec (N.E.d.l.M.-S.); giovanna.moran@ikiam.edu.ec (G.M.-M.); 3 Laboratory of Microbial Biomolecules, Department of Biochemical and Pharmaceutical Technology, University of São Paulo, São Paulo 05508-000, Brazil; 4 Centro Jambatu de Investigación y Conservación de Anfibios, Fundación Jambatu, San Rafael, Quito 171102, Ecuador; 5 School of Pharmacy, University of Reading, Reading RG6 6UB, UK

**Keywords:** toxicity, synthetic antimicrobial peptides, amphibian skin secretion peptides, proline alpha-helix kink

## Abstract

**Background/Objectives**: The increasing antimicrobial resistance is a current human health threat, which has stimulated research on new biologically active molecules against infections caused by microorganisms resistant to conventional therapies. Antimicrobial peptides (AMPs) from amphibian skin secretions have generated great interest in tackling this problem due to their antibacterial, antifungal, antiprotozoal, wound-healing, and even anticancer properties. In Ecuador, there are still unexplored endemic amphibian species as a source of new AMPs, such as *Callimedusa ecuatoriana*. In this study, we report a novel peptide derived from the skin secretion of *Callimedusa ecuatoriana* identified by molecular cloning of the mRNA precursor. The functional analysis demonstrated that it lacks antimicrobial activity due to its alpha-helix kink structure. **Methods**: Inspired by the native structure of PTR-CE1, we designed and synthesized two analogs (PTR-CE1a and PTR-CE1b) to adopt a complete α-helix secondary structure, a conformation often associated with antimicrobial activity. In silico tools were used to predict the peptide activity, which was confirmed by experimental findings. **Results**: Both analogs displayed higher activity than the native peptide, even against the ampicillin-resistant bacterial strain. While PTR-CE1b showed Minimum Inhibitory Concentration (MIC) values of 26.62–212.99 μM and 24.36% of hemolytic activity at 26.62 μM, PTR-CE1a displayed a more potent broad-spectrum activity against all the microorganisms, with MIC values of 3.02–12.06 μM and hemolytic activity of 7.5% at 3.02 μM. **Conclusions**: This study demonstrates the importance of the α-helix structure for antimicrobial activity in *C. ecuatoriana* PTR-CE1 analogs and highlights the potential of unexplored biological and molecular diversity in endemic species of Ecuador to provide novel templates for peptide design.

## 1. Introduction

Antimicrobial resistance (AMR) has emerged as a critical global public health problem [1]. The increase in multidrug-resistant bacteria severely limits our ability to treat infections, leading to increased mortality, particularly in hospital settings. Alarmingly, projections suggest AMR could become the leading cause of death by 2050, claiming over 10 million lives annually [2]. For this reason, there is an urgent need to develop innovative approaches to confront this problem [3].

In this context, amphibian skin secretions have gained attention in antibiotic discovery programs as valuable sources of novel antimicrobial agents. These secretions are a rich natural reservoir of novel antimicrobial peptides (AMPs), which constitute a key component of their innate defense system against predators and pathogens [4,5]. Beyond their antimicrobial properties, these bioactive molecules demonstrate a broad range of therapeutic activities, including diverse properties such as analgesic [6], anti-inflammatory [7], antidiabetic [8], anticancer [9,10], wound-healing [11,12], antioxidant [13], hemostasis effectors [14], and antimicrobial [12,15,16]. This illustrates the potential of peptides as versatile candidates for drug discovery and therapeutic development.

Noticeably, amphibians contribute nearly 60% of all antimicrobial peptides (AMPs) cataloged in the Antimicrobial Peptide Database (APD; ~1900 of 3306 entries) [5,17] (APD, https://aps.unmc.edu, accessed on 1 December 2024), underscoring their pharmaceutical potential [18]. Among amphibian families, Phyllomedusidae exhibit exceptional AMP diversity, comprising eight genera (*Agalychnis*, *Callimedusa*, *Cruziohyla*, *Hylomantis*, *Phasmahyla*, *Phrynomedusa*, *Phyllomedusa*, and *Pithecopus*). While *Phyllomedusa* has been extensively studied (>110 described AMPs) [5,19], *Callimedusa* remains largely unexplored, with only seven reported AMPs [20,21,22,23,24]. This study focuses on *Callimedusa ecuatoriana*, an Ecuadorian endemic species [25], to expand our understanding of this underexplored resource.

Amphibian AMPs represent compelling candidates against drug-resistant pathogens due to their unique mechanism of action [3,26]. Unlike conventional antibiotics, AMPs typically disrupt plasma membrane integrity or target intracellular components and exhibit low propensity for resistance development [27,28]. However, clinical applications face challenges such as limited bioactivity, host toxicity, and proteolytic instability [29,30,31,32,33]. Peptide engineering emerges as a powerful solution to these limitations, enabling the design of synthetic analogs with enhanced antimicrobial properties and reduced toxicity [34,35,36].

Here, we characterize PTR-CE1, a novel Picturin peptide isolated from *C. ecuatoriana* skin secretions. Using this natural peptide as a template, we designed two α-helix-stabilized analogs to investigate how secondary structure influences antimicrobial activity.

## 2. Results

### 2.1. Molecular Cloning of cDNA Encoding PTR-CE1 Precursor

**Figure 1:**
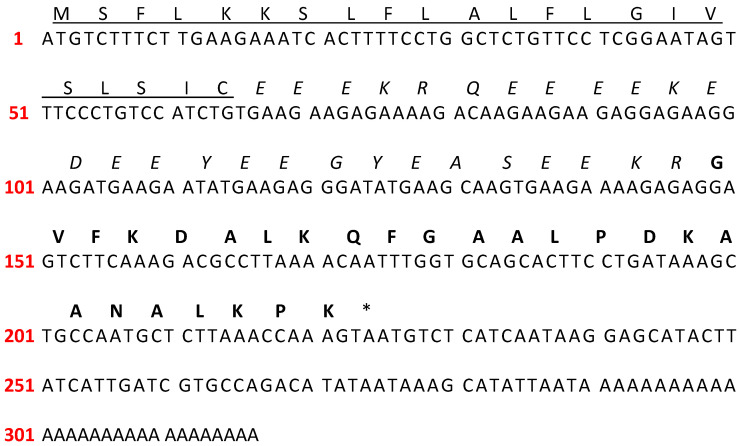
Nucleotide and translated open-reading frame (ORF) amino acid sequence of the sense strand of cloned cDNA encoding PTR-CE1 (OQ438429). Underlined: putative signal peptide; italic: acidic spacer; bold: mature peptide; asterisks: stop codon.

One nucleotide sequence encoding a propeptide was identified from the skin secretion of *C. ecuatoriana* (Figure 1). The translated open-reading frame consisted of 74 amino acids composed of three domains: (1) the signal peptide of 22 amino acids in the N-terminal region, (2) a 27-residue acidic spacer domain containing the KR propeptide processing site, and (3) a 25-mer mature peptide.

The nucleotide sequences and translated ORF amino acid sequences were analyzed using NCBI-BLAST (https://blast.ncbi.nlm.nih.gov/Blast.cgi, accessed on 15 March 2024), revealing a 76.85% similarity with pro-peptide pictuseptin-2 precursor (GeneBank: MW118451.1), 88.49% similarity with pro-peptide boanin-3 (GeneBank: ON703100.1), and 96–98% similarity with pro-peptides from picturin 1, picturin 2, and picturin 3 (GeneBank: MN652613.1, MN652614.1, and MN652615.1, respectively) (Table 1), all isolated from the skin secretion of *Boana picturata* [37]. The alignment with picturins was obtained with more than 93% of query coverage, especially focusing on the signal region and acidic spacer region. These findings suggest that the novel peptide belongs to the picturin family (PTR). Consequently, it was named picturin-CE1 (PTR-CE1) to reflect its origin in *C. ecuatoriana*. The peptide was chemically synthesized for evaluation of its antimicrobial and hemolytic activities.

### 2.2. Peptide Design, Predicted Physicochemical Characteristics, and 3D Models

PTR-CE1 is a 25-mer peptide with a hydrophobicity of 0.236 with a net charge of +3 (Table 2). Compared with other picturin members, PTR-CE1 differs from PTR-2 by a single substitution (Pro15 → Leu) and from PTR-3 by two substitutions (Pro15 → Leu and Lys17 → Gln) (Table 2). These positions include a helix-breaking residue (Pro15) and a charge-altering substitution at position 17, suggesting these differences may influence bioactivity. Previous antimicrobial results demonstrated that PTR-1, PTR-2, and PTR-3 inhibited Gram-positive and Gram-negative bacteria [37].

Based on this information, two analogs were designed using the natural AMP identified in this study to investigate the roles of proline and to increase net charge. PTR-CE1a incorporates four substitutions (Asp5 → Lys, Pro15 → Leu, Asp16 → Arg, and Lys17 → Leu), increasing net charge and restoring the continuous alpha-helix (Table 2). PTR-CE1b is a 23-mer peptide, lacking both proline residues to restore continuous helicity (Table 2). These modifications alter hydrophobicity, peptide length, and net charge, parameters known to influence antimicrobial potency and selectivity. For example, PTR-CE1a and PTR-CE1b have a net charge of +7 and +4, respectively, while the net charge of the native peptide is +3. Both analogs, PTR-CE1a and PTR-CE1b, were C-terminally amidated to reduce proteolytic susceptibility and increase the net charge.

**Figure 2:**
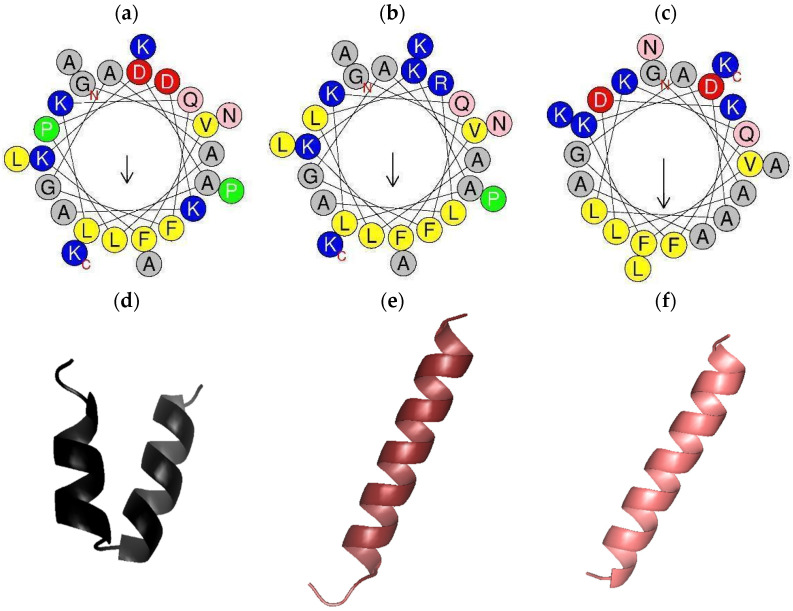
Helical wheel plots and 3D models of PTR-CE1 and their analogs. (**a**) Helical wheel plot of (**a**) PTR-CE1, (**b**) PTR-CE1a, (**c**) PTR-CE1b, and predicted 3D models of (**d**) PTR-CE1, (**e**) PTR-CE1a, (**f**) PTR-CE1b. Residue color code in the helical model: yellow = hydrophobic non-polar, gray = uncharged, blue = polar, red = acid, pink = unladen polar, and green = Pro. Arrows detail a hydrophobic face.

The helical wheel plots demonstrated that peptides are amphipathic (Figure 2). PTR-CE1a exhibited a more pronounced hydrophobic face compared to PTR-CE1 and PTR-CE1b. The three-dimensional structure models obtained using I-TASSER revealed that PTR-CE1a and PTR-CE1b form a single α-helix, whereas PTR-CE1 adopts two α-helices in its structure due to the presence of a proline residue.

### 2.3. In Silico Bioactivity Predictions and Molecular Docking

In silico results showed that PTR-CE1 and its analogs probably have antimicrobial effects (Table 3). Furthermore, HemoPI-2 and ToxinPred analyses predicted that all peptides could induce low hemolysis and are not toxic (Table 3).

**Figure 3:**
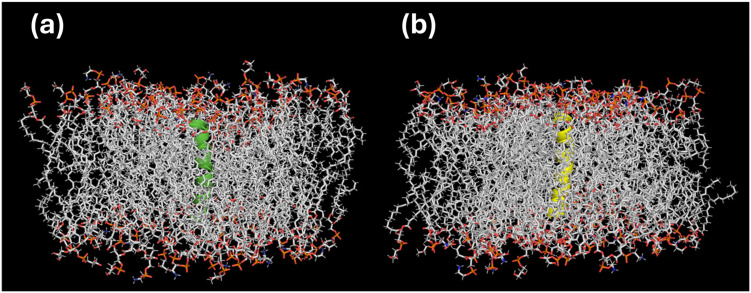
Docking interactions of (**a**) PTR-CE1a (green color) and (**b**) PTR-CE1b (yellow color) with the bacterial cell membrane.

Molecular docking analysis revealed a favorable interaction and affinity between the peptide analogs and the bacterial membrane (Figure 3). PTR-CE1a exhibited a score of −8.4 kcal/mol, and PTR-CE1b showed a score of −4.7 kcal/mol. Considering these in silico data, the peptide analogs were synthesized, and the in vitro bioactivity was evaluated.

### 2.4. Synthesis and Characterization of Peptides

**Figure 4:**
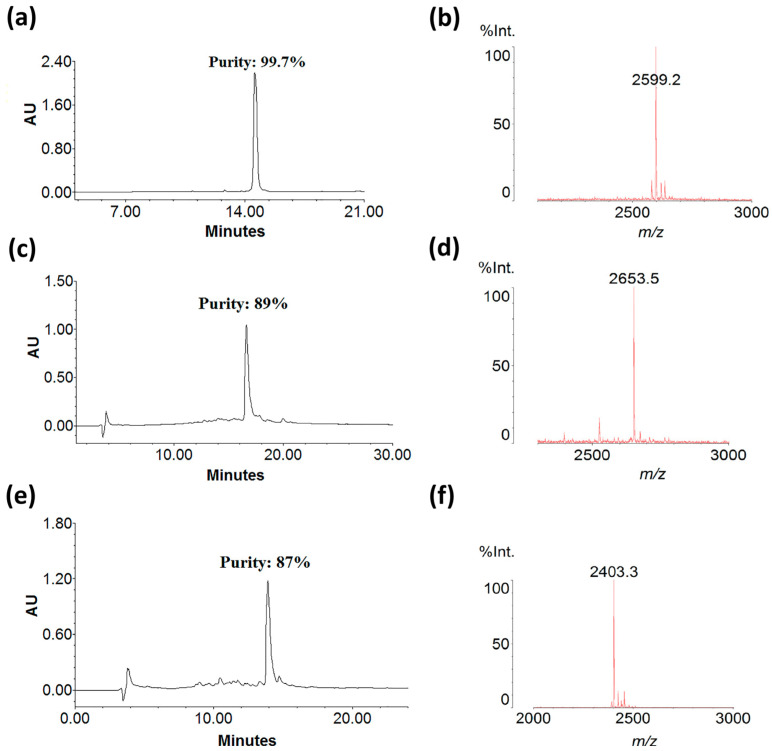
Relative purity and mass spectra of peptides obtained by RP-HPLC and MALDI TOF MS. (**a**) PTR-CE1 purity (99.7%). (**b**) Monoisotopic mass/charge of PTR-CE1 (2599.2 Da). (**c**) PTR-CE1a purity (89%). (**d**) Monoisotopic mass/charge of PTR-CE1a (2653.5 Da). (**e**) PTR-CE1b purity of PTR-CE1b (87%). (**f**) Monoisotopic mass/charge of PTR-CE1b (2403.3 Da).

Chromatography profiles showed that all crude synthetic peptides had >80% purity, and the mass spectrum corroborated the peptide identity (Figure 4), based on the theoretical mass (Table 2).

### 2.5. Antimicrobial Activity of PTR-CE1 and Its Analogs

The antimicrobial activity of the three peptides was evaluated against *Escherichia coli* ATCC 25922, *Staphylococcus aureus* ATCC 29213, *Candida albicans* ATCC 10231, and the ampicillin-resistant strains of *Bacillus subtilis*, *Klebsiella pneumoniae*, and *Pseudomonas aeruginosa*. The native peptide, PTR-CE1, showed no antimicrobial or antifungal activity (Table 4), contrary to in silico predictions. This result highlights the importance of experimental validation to confirm the predicted bioactivity of peptides. On the other hand, the peptide analogs displayed broad-spectrum antimicrobial properties (Table 4) in agreement with the in silico prediction (Table 3). PTR-CE1a emerged as the most potent analog, exhibiting growth inhibition against all tested microorganisms (MIC = 3.02–12.06 μM), including ampicillin-resistant strains. Furthermore, this peptide displayed broad-spectrum antimicrobial activity against Gram-positive bacteria, Gram-negative bacteria, and yeast.

PTR-CE1b showed antimicrobial activity against all bacteria tested except *S. aureus*, including ampicillin-resistant isolates of *E. coli*, *K. pneumoniae*, and *B. subtilis* (MIC = 26.62–53.25 μM) (Table 4). In contrast, its activity against *C. albicans* was markedly weaker (MIC = 212.99 μM).

### 2.6. Hemolytic Activity of Peptides

**Figure 5:**
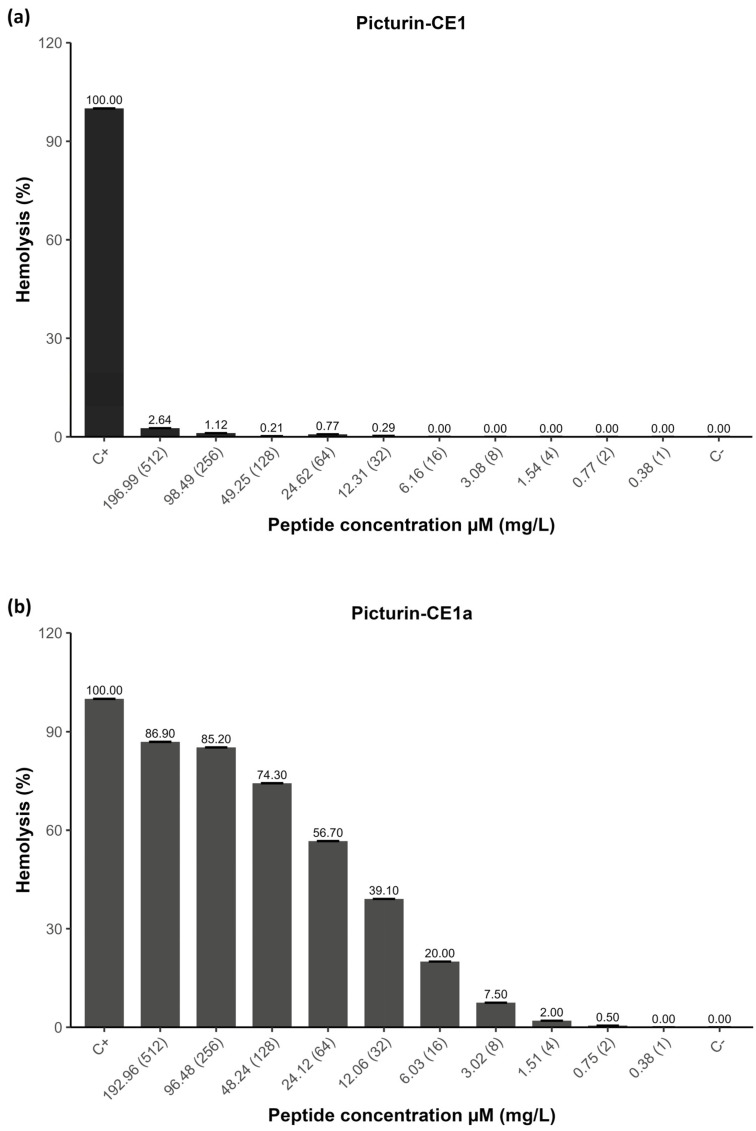

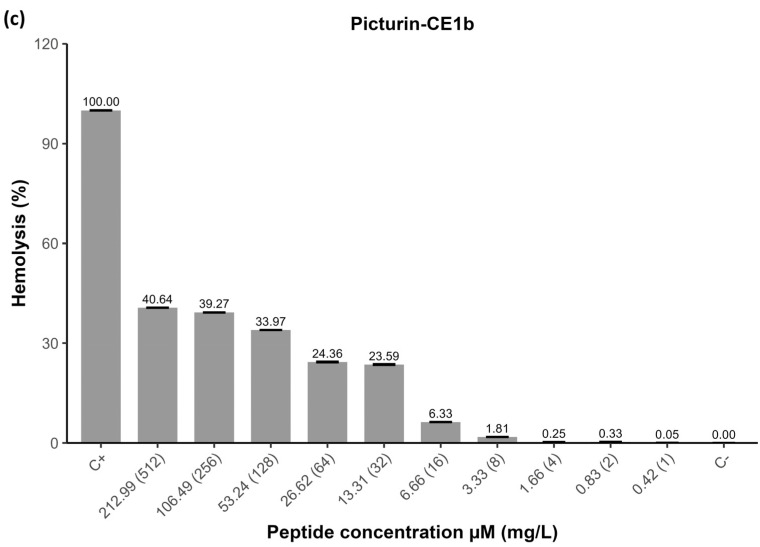
Hemolytic activity of (**a**) PTR-CE1, (**b**) PTR-CE1a, and (**c**) PTR-CE1b. PBS 1X was used as a negative control, and Triton X-100 (2% *v*/*v*) was used as a positive control.

PTR-CE1 exhibited only 2.64% of hemolytic activity at the highest tested peptide concentration (196.99 μM). In contrast, both analogs displayed higher hemolytic activity than the native peptide. PTR-CE1a showed 7.5–39.1% hemolytic activity at the MICs against the tested pathogens (MIC = 3.02–12.06 μM), whereas PTR-CE1b induced 24.36–40.64% hemolytic activity at the MIC (26.62–212.99 μM) (Figure 5).

## 3. Discussion

Several studies have shown the abundance of antimicrobial peptides (AMPs) in frog skin secretions [5,8,43]. However, hundreds of species remain unstudied, especially in megadiverse countries like Ecuador [44]. In this study, molecular cloning identified a novel peptide precursor (PTR-CE1) from the skin secretion of the endemic frog *Callimedusa ecuatoriana*. The signal peptide and the acidic spacer of the PTR-CE1 precursor showed a high similarity with those of picturins [37], a peptide family previously described in *Boana picturata* (Table 1). In fact, the mature peptide was 92–96% similar to picturin peptides, justifying its inclusion in this family. The mature PTR-CE1 peptide is composed of 25 amino acid residues and is the first skin secretion peptide reported from this endemic Ecuadorian frog species.

All previously reported members of the picturin family have antibacterial properties, such as PTR-1, PTR-2, and PTR-3 [37]. PTR-1 showed the highest activity against *E. coli* (MIC = 24.80 μM) and *S. aureus* (MIC = 198.37 μM). However, PTR-CE1 did not inhibit the growth of the tested bacteria. PTR-1 and PTR-CE1 share some characteristics, such as a cationic charge (+3) and similar hydrophobicity values (0.236–0.271). However, their primary sequence differs in the amino acid composition: at position 10 (Leu/Phe) and at position 15 (Leu/Pro). Likewise, PTR-2 has an amino acid substitution at position 15 (Leu/Pro) compared to the natural peptide PTR-CE1.

Furthermore, bioinformatic predictions demonstrated that the proline residue in PTR-CE1 induces a helix–kink–helix motif in its three-dimensional model. This structural alteration is likely responsible for the lack of antimicrobial effects, as several studies have reported that such kinks reduce the affinity between AMPs and bacterial membranes, leading to a loss of antimicrobial activity [45,46,47,48].

Conversely, numerous studies indicate that the formation of a continuous α-helix is strongly correlated with bioactivity of AMPs [49,50,51,52]. To test this hypothesis, we designed two peptide analogs using PTR-CE1 as a scaffold. In silico results showed that both analogs, PTR-CE1a and PTR-CE1b, form a continuous α-helix in their 3D models. Furthermore, in silico CAMPR3 predictions yielded values close to 1, indicating that the designed peptides are likely antimicrobial. HemoPI-2 values were slightly higher than those for PTR-CE1, suggesting low hemolytic activity (Table 3) [53]. Antimicrobial assays successfully confirmed the bioactivity of these peptide analogs. PTR-CE1a displayed broad-spectrum activity against all tested microorganisms, including ampicillin-resistant isolates, while PTR-CE1b was active against the same microorganisms except for *S. aureus*.

A clear difference in potency was observed between the two peptides. The MIC of PTR-CE1a ranged from 3.02 to 12.06 μM, whereas the MIC of PTR-CE1b ranged from 106.5 to 212.99 μM, making PTR-CE1a as potent as commercial antimicrobial drugs and several designed peptides from amphibians [5,39,41,54,55]. This difference in MIC values is probably due to the higher net charge of PTR-CE1a (+7 vs. +4). These findings align with several studies demonstrating that peptides with increased cationicity exhibit enhanced antimicrobial effects compared to the original parent peptide [39,55,56].

Many frog-derived AMPs target the bacterial membrane [27,28]. Given the cationic and hydrophobic nature of PTR-CE1a and PTR-CE1b, these peptides likely inhibit pathogens via membranolytic effects. Indeed, molecular docking results showed that both peptide analogs interacted with and were embedded within a mimetic bacterial membrane, suggesting potential membrane damage.

Although these analogs show antimicrobial activity, their toxicity constitutes a major barrier to further development as therapeutic candidates. Unlike PTR-CE1, PTR-CE1a showed potent antimicrobial effects but significant hemolytic activity at the minimum inhibitory concentrations. In contrast, PTR-CEb showed lower hemolytic activity than PTR-CEa but also weaker antimicrobial activity. The ratio between the MIC and hemolytic activity is known as the selectivity index (SI), and an SI greater than 10 is generally recommended for therapeutic potential [57]. Therefore, these analogs are far from being ideal candidates for therapeutic use [58]. Nevertheless, our results demonstrate that generating analogs is a valuable strategy for understanding the physicochemical parameters and structural features required for antimicrobial activity, which can sometimes yield promising molecules with SI higher than 140 [59,60].

In summary, a novel peptide characterized from *C. ecuatoriana* that lacked antimicrobial activity was used as a template to design peptide analogs with potent antimicrobial activity. Their activity and significant toxicity are primarily attributed to their alpha-helical structure, increased hydrophobicity, and cationicity. Further design strategies are required to reduce toxicity. Therefore, our study highlights that nature-based resources represent an important source of peptides with the potential for the development of bio-inspired drugs for biomedical or biotechnological applications.

## 4. Materials and Methods

### 4.1. Collection of Callimedusa ecuatoriana Skin Secretions

One adult specimen collected from Cordillera del Cóndor, Morona Santiago Province, and two captive-born subadult specimens were provided in 2019 by Centro Jambatu for Amphibian Research and Conservation (Ecuador). Briefly, cutaneous secretions were obtained through gentle non-invasive massages to stimulate the release of skin defensive exudates from the skin granular glands. Thereafter, the secretions were collected using distilled water, immediately frozen at −80° C, lyophilized in a VirTis Benchtop Pro Freeze Dryer (Sp Scientific, Warminster, PA, USA), and stored at −20 °C [61]. Following the procedure, all specimens were returned to their terrariums without apparent harm after the process.

### 4.2. “Shotgun” Cloning of the Novel Peptide Precursor from C. ecuatoriana Skin Secretion-Derived cDNA Library

The poly-adenylated (poly A) mRNA from *C. ecuatoriana* skin secretion was obtained from 5 mg of lyophilized secretion dissolved in 1 mL of lysis/binding buffer using Dynabeads^®^ mRNA DIRECT™ kit (Dynal Biotec, Merseyside, UK). The isolated mRNA was reverse transcribed to build a cDNA library using GoScript™ Reverse Transcription System and 3′CDS primer (5′-AAGCAGTGGTATCAACGCAGAGTACT30VN-3′; V = A + C + G, N = A + T + C + G) (20 μM). Then, 3′-RACE PCR was performed to obtain *C. ecuatoriana* precursor peptide sequences using forward primer (5′-GACCAAAGATGTCWTTCTTGAAGAAAT-3′), designed from a highly conserved N-terminal opioid peptide of *Agalychnis dacnicolor* (GenBank AJ005443.1), and reverse Nested Universal Primer (NUP, 5′-AAGCAGTGGTATCAACGCAGAGT-3′). PCR products were analyzed by 2% agarose gel electrophoresis and purified using the PureLink™ PCR Purification Kit system (Invitrogen™, Carlsbad, CA, USA). The concentration and purity of DNA were verified with a Thermo Scientific™ NanoDrop™ One Microvolume UV-VIS spectrophotometer (Thermofisher, Waltham, MA, USA) and cloned using a pGEM^®^-T Easy vector system (Promega Corporation, Southampton, UK). Finally, the nucleotide sequence of encoded biosynthetic precursors of antimicrobial peptides was obtained by Sanger sequencing (Universidad de las Americas, Laboratory Services, Quito, EC, Ecuador).

### 4.3. Identification of the Novel Peptide from C. ecuatoriana Skin Secretion

The nucleotide sequences were analyzed and translated into amino acid sequences through MEGA 11 [62]. The basic local alignment was performed in BLAST/n and BLAST/p of the National Center for Biotechnology Information (NCBI) [63].

### 4.4. Computer-Aided Peptide Design, In Silico Predictions, and Molecular Docking

PTR-CE1 (PTR-CE1) was used as a template to design two analog peptides with enhanced antimicrobial activity. This design was supported by predictors of in silico bioactivity and toxicity. CAMPR3 was used to screen the antimicrobial effect, and HemoPI-2 to evaluate the hemolytic activity [53,64]. Additionally, the secondary structure prediction was carried out in the Self-Optimized Prediction Method from Alignment (SOPMA, https://npsa-pbil.ibcp.fr/cgi-bin/npsa_automat.pl?page=/NPSA/npsa_sopma.html, accessed on 15 January 2024) [65], and the helical wheel model of the peptides was performed using HeliQuest V2 [66]. This last tool and the Bachem Peptide Calculator were used to obtain physicochemical properties, while the theoretical mass was calculated on Peptide Mass Calculator V3.2 [67]. Finally, the three-dimensional structure of PTR-CE1 and its analogs was obtained using the I-TASSER server [68] and visualized by Pymol V3.1 [69].

Docking analysis was performed to explore the possible membranolytic effect of the peptide analogs. A bacterial membrane, composed of POPG (1-palmitoyl-2-oleoyl-sn-glycero-3-phosphoglycerol) and POPE (1-palmitoyl-2-oleoyl-sn-glycero-3-phosphoethanolamine) lipids in a ratio of 3:1, was constructed using the CHARMM-gui server V3.8 [70]. The peptide structure obtained in the previous step was used. Finally, the software Autodock Vina V1.2.X was employed in molecular docking [71]. The interaction energy between the peptide and membrane was expressed as affinity (kcal/mol).

### 4.5. Solid-Phase Peptide Synthesis (SPPS)

PTR-CE1 and two analogs were chemically synthesized using an automatic microwave peptide synthesizer (CEM Corporation, Matthews, NC, USA), with the solid-phase Fmoc (9-fluorenyl-methoxycarbonite) strategy. Cl-TCP (Cl) ProTide Resin (CEM, USA) was used to synthesize the acidic PTR-CE1 and Fmoc Rink Amide resin (LL) (CEM, USA) for synthesizing their analogs containing a C-terminal amidated. *N*,*N*′-dimethylformamide (DMF) was the main solvent, and 20% piperidine was used as the deprotection reagent, while Oxyma 1M and *N*,*N*′-diisopropylcarbodiimide (DIC) 1M were used as activators in the coupling process. To cleave the resin–peptide bond, the synthesized product was incubated in the microwave system at 38 °C for 30 min with a cleavage cocktail of trifluoroacetic acid (TFA), triisopropyl silane (TIPS), 3,6-dioxa-1,8-octanedithiol (DODT), and water (92.5/2.5/2.5/2.5 *v*/*v*/*v*/*v*). Synthetic peptides were collected and washed after overnight precipitation using cold diethyl ether and centrifugation (5000 rpm for 15 min). Subsequently, these products were freeze-dried using a Virtis BenchTop Pro (SP Scientific) in vacuum conditions at −80 °C and stored at −20 °C. The peptide identity was confirmed by AXIMA Confidence MALDI TOF MS (Shimadzu, Columbia, MD, USA) in positive detection mode using α-Cyano-4-hydroxycinnamic acid (CHCA) as a matrix (10 mg/mL). Finally, the purity was analyzed by reverse-phase high-performance liquid chromatography (RP-HPLC) applying a linear gradient of 10% buffer (0.05% TFA, 99.95% water) to 100% buffer (99.95% CAN, 0.05% TFA) for 240 min at a 1 mL/min flow rate, with a four-pump chromatograph (Waters, Milford, MA, USA) coupled to a C_18_ Jupiter column (250  ×  4.6 mm, 300 Å, 5 µm) and a UV–VIS detector at 214 nm [37,43].

### 4.6. Minimum Inhibitory Concentration (MIC), Minimum Bactericidal Concentration (MBC), and Minimum Fungicidal Concentration (MFC)

The minimum inhibitory concentration (MIC), minimum bactericidal concentration (MBC), and minimum fungicidal concentration (MFC) of the synthetic peptides against *Escherichia coli* ATCC 25922, *Staphylococcus aureus* ATCC 25923, *Bacillus subtilis* (ampicillin-resistant), *Klebsiella pneumoniae* (ampicillin-resistant), *Pseudomonas aeruginosa* (ampicillin-resistant), and *Candida albicans* ATCC 10231 were determined according to the protocol of Proaño-Bolaños et al. [43]. The microorganisms were cultured in Mueller–Hinton broth (MHB) to reach log phase growth (10^8^ CFU/mL for bacteria and 10^6^ CFU/mL for yeast), and sterile MHB was used to dilute the bacterial culture concentration to 10^6^ CFU/mL. Serial dilutions of peptides (1, 2, 4, 8, 16, 32, 64, 128, 256, 512 × 10^2^ mg/L) were prepared in DMSO. Then, 2 μL of peptide dilutions was mixed with 198 μL of each microorganism suspension in a 96-well microplate. MHB sterile and DMSO instead of the synthetic peptide were used as controls. Five replicates of each concentration and control were performed, and the assay was repeated three times. The microplates were incubated at 37 °C for 18 h. MIC was determined by measuring the microorganism growth at 600 nm using a GloMax^®^ microplate reader (Promega Corporation, Madison, WI, USA). MBC and MFC were registered considering the lowest concentration without any microorganism growth after 10 μL of each concentration was cultured on Muller–Hinton Agar (MHA) and incubated overnight at 37 °C.

### 4.7. Hemolytic Activity

The hemolytic activity of the peptides against human red blood cell was evaluated using a 4% (*v*/*v*) suspension of erythrocytes in sterile phosphate-buffered saline (PBS) 1X. Later, 200 μL of this solution was incubated with 200 μL of serial peptide dilution (1, 2, 4, 8, 16, 32, 64, 128, 256, 512 mg/L) at 37 °C for 2 h. PBS instead of peptide was used as a negative control, and a PBS solution with Triton X-100 (2% *v*/*v*) was added as a positive control. Subsequently, the samples were centrifuged at 1000× *g* for 5 min, and the supernatant was transferred to 96-well microplates. Lysis of blood cells was quantified in a GloMax^®^ plate reader (Promega, USA) at 560 nm. The percentage of hemolysis was calculated as follows:

% Hemolysis = (A − A0)/(Ax − A0) × 100%, where A = OD for the sample, Ax = OD for the positive control, and A0 = OD for the negative control [72].

## Figures and Tables

**Table 1:** Domain structure comparison of PTR-CE1 with other similar peptide precursors.

Peptide	Signal Peptide																																							
									1																															
Picturin-CE1										M	S	F	L	K	K	S	L	F	L	A	L	F	L	G	I	V	S	L	S	I	C									
Picturin-2										M	S	F	L	K	K	S	L	F	L	V	L	F	L	G	I	V	S	L	S	I	C									
Picturin-3										-	-	-	-	-	K	S	L	F	L	V	L	F	L	G	I	V	S	L	S	I	C									
Picturin-1										-	-	-	-	-	-	S	L	F	L	V	L	F	L	G	I	V	S	L	S	I	C									
Boanin-3										M	T	F	G	K	K	S	L	F	L	V	L	F	L	G	M	V	S	L	S	I	C									
Pictuseptin-2										M	S	F	L	K	K	S	L	F	L	V	L	F	L	G	I	V	S	L	S	I	C									
		**Acidic spacer**																																						
					23																																			
Picturin-CE1						E	E	E	K	R	-	-	Q	E	E	E	E	K	E	D	E	E	Y	E	E	G	Y	E	A	S	E	E	K	R						
Picturin-2						E	E	E	K	R	-	-	Q	E	E	E	E	K	E	D	E	E	Y	E	E	G	Y	E	A	S	E	E	K	R						
Picturin-3						E	E	E	K	R	-	-	Q	E	E	E	E	K	E	D	E	E	Y	E	E	G	Y	E	A	S	E	E	K	R						
Picturin-1						E	E	E	K	R	-	-	Q	E	E	E	E	K	E	D	E	E	Y	E	E	G	Y	E	A	S	E	E	K	R						
Boanin-3						Q	D	E	K	R	-	E	E	E	E	E	E	K	E	E	E	E	Y	E	E	G	N	E	E	H	K	E	K	R						
Pictuseptin-2						E	E	E	K	K	Q	A	E	E	E	E	E	K	Q	E	E	Q	Y	D	Q	E	N	E	E	Y	K	E	K	R						
	**Mature peptide**																																**Accession number**							
	52																																							
Picturin-CE1		-	-	-	G	V	F	K	D	A	L	K	Q	F	G	A	A	L	P	D	K	A	A	N	A	L	K	P	K	*			OQ438429.1							
Picturin-2		-	-	-	G	V	F	K	D	A	L	K	Q	F	G	A	A	L	L	D	K	A	A	N	A	L	K	P	K	*			MN652614.1							
Picturin-3		-	-	-	G	V	F	K	D	A	L	K	Q	F	G	A	A	L	L	D	Q	A	A	N	A	L	K	P	K	*			MN652615.1							
Picturin-1		-	-	-	G	V	F	K	D	A	L	K	Q	L	G	A	A	L	L	D	K	A	A	N	A	L	K	P	K	*			MN652613.1							
Boanin-3		-	F	L	G	A	L	F	A	I	G	K	A	I	G	K	A	I	L	P	L	A	V	K	A	F	N	P	Q	H	*		ON703100.1							
Pictuseptin-2		G	F	L	D	T	L	K	N	I	G	K	T	V	G	-	-	-	-	G	I	A	L	N	V	L	T	G	*				MW118451.1							

Light gray: Conserved sites in all sequences. Dark gray: Similar sites only in the mature peptide region. Black: Unique amino acid in mature peptide region of PTR-CE1. *: Stop codon.

**Table 2:** Physicochemical characteristics of PTR, PTR-CE1, and their analogs.

Peptide	Sequence																										#Aas	Alpha Helix %	Hydrophobicity <H>	Hydrophobic Moment <mH>	Net Charge Z	Theoretical Mass	Ref.
	h	h	h	h	h	h	h	h	h	h	h	h	h	c	c	h	h	h	h	h	h	h	c	t	t								
PTR-CE1	G	V	F	K	D	A	L	K	Q	F	G	A	A	L	P	D	K	A	A	N	A	L	K	P	K		25	80	0.236	0.506	3	2599.07	
	h	h	h	h	h	h	h	h	h	h	h	h	h	h	h	h	h	h	h	h	h	h	c	t	t								
PTR-CE1a	G	V	F	K	K	A	L	K	Q	F	G	A	A	L	L	R	L	A	A	N	A	L	K	P	K	a	25	88	0.364	0.462	7	2653.30	
	h	h	h	h	h	h	h	h	h	h	h	h	h	h	h	h	h	h	h	h	h	h	h										
PTR-CE1b	G	V	F	K	D	A	L	K	Q	F	G	A	A	L	D	K	A	A	N	A	L	K	K	a			23	100	0.193	0.468	4	2403.85	
	h	h	h	h	h	h	h	h	h	h	h	h	h	h	h	h	h	h	h	h	h	h	c	t	t								
PTR-1	G	V	F	K	D	A	L	K	Q	L	G	A	A	L	L	D	K	A	A	N	A	L	K	P	K		25	88	0.271	0.306	3	2581.10	[37]
	h	h	h	h	h	h	h	h	h	h	h	h	h	h	h	h	h	h	h	h	h	h	c	t	t								
PTR-2	G	V	F	K	D	A	L	K	Q	F	G	A	A	L	L	D	K	A	A	N	A	L	K	P	K		25	88	0.275	0.309	3	2615.12	[37]
	h	h	h	h	h	h	h	h	h	h	h	h	h	h	h	h	h	h	h	h	h	h	c	t	t								
PTR-3	G	V	F	K	D	A	L	K	Q	F	G	A	A	L	L	D	Q	A	A	N	A	L	K	P	K		25	88	0.306	0.326	2	2615.07	[37]

a = amidation, h = alpha helix, t = beta turn, c = random coil.

**Table 3:** In silico screening of antimicrobial effect, hemolytic activity, and toxicity of PTR-CE1 and its analogs.

Peptide	CAMPR3 (SVM *)	HemoPI-2	ToxinPred
	Antimicrobial		
PTR-CE1	0.915	0.52	Non-toxin
PTR-CE1a	0.994	0.58	Non-toxin
PTR-CE1b	0.908	0.54	Non-toxin

* SVM = Support Vector Machine.

**Table 4:** Minimum inhibitory concentration (MIC) and minimum bactericidal concentration (MBC) of PTR-CE1 and its analogs.

	MIC μM (mg/L)						MBC μM (mg/L)						Ref.
Synthetic Peptide	*E. coli*	*S. aureus*	*C. albicans*	*K. pneumoniae*	*P. aeruginosa*	*Bacillus subtilis*	*E. coli*	*S. aureus*	*C. albicans*	*K. pneumoniae*	*P. aeruginosa*	*Bacillus subtilis*	
	25922	25923		(AMP-RES)	(AMP-RES)	(AMP-RES)	25922	25923		(AMP-RES)	(AMP-RES)	(AMP-RES)	
				Clinical Isolate	Clinical Isolate	Clinical Isolate				Clinical Isolate	Clinical Isolate	Clinical Isolate	
Picturin-CE1	>196.99	>196.99	>196.99	>196.99	>196.99	>196.99	>196.99	>196.99	>196.99	>196.99	>196.99	>196.99	
	(>512)	(>512)	(>512)	(>512)	(>512)	(>512)	(>512)	(>512)	(>512)	(>512)	(>512)	(>512)	
Picturin-CE1a	3.02	6.03	12.06	6.03	12.06	3.02	6.03	12.06	>192.96	12.06	48.24	24.12	
	(8)	(16)	(32)	(16)	(32)	(8)	(16)	(32)	(>512)	(32)	(128)	(64)	
Picturin-CE1b	53.25	>212.99	212.99	53.25	26.62	26.62	106.49	>212.99	>212.99	106.49	>212.99	53.25	
	(128)	(>512)	(512)	(128)	(64)	(64)	(256)	(>512)	(>512)	(256)	(>512)	(128)	
* Picturin-1	24.80	198.37	>198.37	ND	ND	ND	>198.37	>198.37	>198.37	ND	ND	ND	[37]
	(64)	(512)	(>512)				(>512)	(>512)	(>512)				
* Picturin-2	48.95	>195.79	>195.79	ND	ND	ND	48.95	>195.79	>195.79	ND	ND	ND	[37]
	(128)	(>512)	(>512)				(128)	(>512)	(>512)				
* Picturin-3	48.98	97.95	>195.91	ND	ND	ND	48.98	>195.91	>195.91	ND	ND	ND	[37]
	(128)	(256)	(>512)				(128)	(>512)	(>512)				
* Pseudin-2	5	20	ND	ND	ND	ND	ND	ND	ND	ND	ND	ND	[38]
[Lys3,10,14,21]													
* Hylin a1-2A	16	2	ND	ND	ND	ND	ND	ND	ND	ND	ND	ND	[39]
* Brevinin-1BYa	20	ND	ND	ND	ND	ND	ND	ND	ND	ND	ND	ND	[40]
[Ser18,Ser24]													
* Alysterin-2a	ND	64	64	ND	ND	ND	ND	ND	ND	ND	ND	ND	[41]
* Kassinatuerin-1	6.25	6.25	25	ND	ND	ND	ND	ND	ND	ND	ND	ND	[42]
[Lys7,Lys18,Lys19]													
* Brevinin-2Ob	4	9	40										
* Ampicillin	46	<11	ND	ND	ND	ND	ND	ND	ND	ND	ND	ND	[12]
* Fluconazole	ND	ND	209	ND	ND	ND	ND	ND	ND	ND	ND	ND	[16]

ND = No data. * MIC values of analog peptides from amphibian skin secretion of other species and commercial drugs were added for comparison.

## Data Availability

The original contributions presented in this study are included in the article.
